# Correction: Concise synthesis and biological activity evaluation of novel pyrazinyl–aryl urea derivatives against several cancer cell lines, which can especially induce T24 apoptotic and necroptotic cell death

**DOI:** 10.1039/d1md90046c

**Published:** 2021-12-16

**Authors:** Jia-Nian Chen, Chu-Ting Chen, Yue-Zhen He, Tai-Sheng Qin, Li Cheng, Ye-Xiang Sun, Kang-Jian Yang, Qi Chen, Chao Yang, Ying Wei

**Affiliations:** State Key Laboratory for Chemistry and Molecular Engineering of Medicinal Resources, Collaborative Innovation Center for Guangxi Ethnic Medicine, School of Chemistry and Pharmaceutical Sciences, Guangxi Normal University Yucai Road 15 Guilin 541004 Guangxi P.R. China cjn288@mailbox.gxnu.edu.cn cctichen@sina.com yzhe226@sina.com qintaisheng@sina.com missliesel@163.com wks72018@sina.com ykj01007@sina.com chen412qi@sina.com yangchao208509@sina.com weiying423@sina.com +86 0773 2120958 +86 0773 2120958

## Abstract

Correction for ‘Concise synthesis and biological activity evaluation of novel pyrazinyl–aryl urea derivatives against several cancer cell lines, which can especially induce T24 apoptotic and necroptotic cell death’ by Jia-Nian Chen *et al., RSC Med. Chem.*, 2021, DOI: 10.1039/d1md00306b.

The authors regret that there were a few errors in compound nomenclature and some other typographical errors in their manuscript, as shown in the Table below:

**Table:** 

1	Page 1, right column, 2 lines from the bottom	‘its receptor vascular’ needs to be corrected to ‘its receptor–vascular’
2	Page 2, right column, lines 7–8	In the sentence ‘we have focused on T24 cell death induced by **5–23** and try to give reasonable explanations…’, ‘try’ needs to be corrected to ‘tried’
3	Page 3, Table 1, compound **5–24**	R needs to be corrected to 4-(*N*,*N*-dimethylamino)phenyl
4	Page 11, right column, line 21	*N*,*N*-Dimethyl needs to be corrected to *N*,*N*-dimethylamino
5	Pages 14 and 15, correction of the following compounds names to remove space before urea	*N*-((3-Chloro-4-fluoro)phenyl)-*N*′-(3-(((3-chloropyrazin-2-yl)amino)methyl)phenyl)urea (**5**–**17**) *N*-((3-Chloro-4-methyl)phenyl)-*N*′-(3-(((3-chloropyrazin-2-yl)amino)methyl)phenyl)urea (**5**–**20**) *N*-((2-Bromo-4-chloro)phenyl)-*N*′-(3-(((3-chloropyrazin-2-yl)amino)methyl)phenyl)urea (**5**–**21**) *N*-((3-Trifluoromethyl)phenyl)-*N*′-(3-(((3-chloropyrazin-2-yl)amino)methyl)phenyl)urea (**5**–**22**) *N*-((4-Trifluoromethyl)phenyl)-*N*′-(3-(((3-chloropyrazin-2-yl)amino)methyl)phenyl)urea (**5**–**23**)
6	Page 15, right column, correction of compound name(as indicated in entry 1)	*N*-(4-(*N*,*N*-Dimethylamino)phenyl)-*N*′-(3-(((3-chloropyrazin-2-yl)amino)methyl)phenyl)urea (**5**–**24**)
7	Page 15, right column, correction of compound name (methanesulfyl to methylsulfonyl)	*N*-((4-Methylsulfonyl)phenyl)-*N*′-(3-(((3-chloropyrazin-2-yl)amino)methyl)phenyl)urea (**5**–**25**)
8	Page 17, left column, in the primer sequences the symbol “-” in the following 4 lines needs to be deleted. In detail, corrections are as follows in lines 11, 8, 4, and 3 from the bottom	5′-CATTTCCTGTTCCCTCTCCA-3′ 5′-AGGAGGCTAATGGGGAGATAGA-3′ 5′-CGACGTGTGGTCTTACGGAGTA-3′ 5′-ACCTCATATCTGTCCTGATGTGATAT-3′

The Royal Society of Chemistry apologises for these errors and any consequent inconvenience to authors and readers.

## Supplementary Material

